# Dynamic Pneumococcal Genetic Adaptations Support Bacterial Growth and Inflammation during Coinfection with Influenza

**DOI:** 10.1128/IAI.00023-21

**Published:** 2021-06-16

**Authors:** Amanda P. Smith, Lindey C. Lane, Tim van Opijnen, Stacie Woolard, Robert Carter, Amy Iverson, Corinna Burnham, Peter Vogel, Dana Roeber, Gabrielle Hochu, Michael D. L. Johnson, Jonathan A. McCullers, Jason Rosch, Amber M. Smith

**Affiliations:** a Department of Pediatrics, University of Tennessee Health Science Center, Memphis, Tennessee, USA; b Department of Biology, Boston College, Chestnut Hill, Massachusetts, USA; c Department of Flow Cytometry, St. Jude Children’s Research Hospital, Memphis, Tennessee, USA; d Department of Oncology, St. Jude Children’s Research Hospital, Memphis, Tennessee, USA; e Department of Infectious Diseases, St. Jude Children’s Research Hospital, Memphis, Tennessee, USA; f Department of Pathology, St. Jude Children’s Research Hospital, Memphis, Tennessee, USA; g The Hartwell Center, St. Jude Children’s Research Hospital, Memphis, Tennessee, USA; h Department of Immunobiology, University of Arizona, Tucson, Arizona, USA; University of Illinois at Chicago

**Keywords:** influenza virus, pneumococcus, genetic adaptation, immune response, metabolism, pathogenesis

## Abstract

Streptococcus pneumoniae (pneumococcus) is one of the primary bacterial pathogens that complicates influenza virus infections. These bacterial coinfections increase influenza-associated morbidity and mortality through a number of immunological and viral-mediated mechanisms, but the specific bacterial genes that contribute to postinfluenza pathogenicity are not known. Here, we used genome-wide transposon mutagenesis (Tn-Seq) to reveal bacterial genes that confer improved fitness in influenza virus-infected hosts. The majority of the 32 genes identified are involved in bacterial metabolism, including nucleotide biosynthesis, amino acid biosynthesis, protein translation, and membrane transport. We generated mutants with single-gene deletions (SGD) of five of the genes identified, SPD1414, SPD2047 (*cbiO1*), SPD0058 (*purD*), SPD1098, and SPD0822 (*proB*), to investigate their effects on *in vivo* fitness, disease severity, and host immune responses. The growth of the SGD mutants was slightly attenuated *in vitro* and *in vivo*, but each still grew to high titers in the lungs of mock- and influenza virus-infected hosts. Despite high bacterial loads, mortality was significantly reduced or delayed with all SGD mutants. Time-dependent reductions in pulmonary neutrophils, inflammatory macrophages, and select proinflammatory cytokines and chemokines were also observed. Immunohistochemical staining further revealed altered neutrophil distribution with reduced degeneration in the lungs of influenza virus-SGD mutant-coinfected animals. These studies demonstrate a critical role for specific bacterial genes and for bacterial metabolism in driving virulence and modulating immune function during influenza-associated bacterial pneumonia.

## INTRODUCTION

Bacterial pathogens often complicate influenza virus infections, causing increased morbidity and mortality, and Streptococcus pneumoniae (pneumococcus) is one of the leading pathogens that has presented as a risk factor for hospitalization, severe disease, and mortality during influenza epidemics and pandemics (1–7). There is considerable genetic diversity within and between pneumococcal serotypes, and laboratory and clinical studies suggest that specific strains are preferentially promoted in influenza virus-infected hosts (8–10). Despite the clinical importance of the synergy between influenza viruses and pneumococci, a systematic investigation into bacterial factors that contribute to coinfection disease severity has yet to be performed.

During influenza A virus (IAV) coinfection with pneumococcus, bacteria are able to grow rapidly, the viral burden increases, and significant inflammation amasses. The host-pathogen interplay is complex, with numerous factors contributing to pathogen growth and host disease. Several studies have investigated the impact of viral virulence factors (e.g., reduced epithelial integrity, PB1-F2, and viral neuraminidase [NA]), bacterial virulence factors (e.g., bacterial NA, enhanced attachment, and sialic acid catabolism), and host immune responses (e.g., cytokine storm and cell dysfunction and death) on the pathogenicity of bacterial coinfections during influenza virus infection (11–17). The incidence and severity of coinfection is, in part, a function of the detrimental effects that influenza virus infection has on key immune responses (e.g., alveolar macrophage [AMΦ] depletion and dysfunction [18–23], neutrophil and inflammatory macrophage dysfunction [23–32], and abundant production of proinflammatory cytokines [12–17, 33]). However, the role that single genes play in pathogenicity during coinfection remains unclear.

Pneumococci, in particular, are highly adaptable, altering their gene expression and metabolic functions to adjust to multiple host niches during infection, and several genes have been identified as regulators of transmissibility and disease severity during primary pneumococcal infection (34–45). Select pneumococcal virulence factors have been investigated in the context of IAV infection (e.g., sialic acid catabolism) (46–49), but systematic genomic screens have not yet been employed to assess pneumococcal adaptations during IAV-pneumococcus coinfection. Genome-wide screens have been used to identify influenza virus-induced changes during Haemophilus influenzae coinfection (50), which included changes in purine biosynthesis, amino acid metabolism, iron homeostasis, and cell wall synthesis (50). Genomic screens assessing pneumococcal adaptations of the TIGR4 strain in hosts with sickle cell anemia suggested that genes involved in purine biosynthesis, complement function, and iron acquisition were of importance (51). Given these findings and the similarities in bacterial metabolic adaptations under various host pressures, understanding how bacterial genes influence influenza virus-pneumococcus coinfection is imperative.

Here, we used genome-wide transposon insertion sequencing (Tn-Seq) (52) to investigate all nonessential pneumococcal genes that modulate disease severity and immune responses. Doing so could help establish important species- and strain-dependent and -independent mechanisms amenable to targeting with therapeutics. Our Tn-Seq screen revealed a total of 32 genes, some with known metabolic functions, that confer bacterial fitness during influenza virus-pneumococcus coinfection compared to primary bacterial infection. Interestingly, there were time-dependent differences. To determine how select genes affect pathogenicity and host immune responses, we generated 5 single-gene-deletion (SGD) mutants in pneumococcal strain D39 (D39Δ*cbiO1* [SPD2047], D39Δ*purD* [SPD0058], D39Δ*1414*, D39Δ*1098*, and D39Δ*proB* [SPD0822]). Lethality was significantly reduced or eliminated during infection despite high bacterial loads in the lungs and blood. This was concurrent with significant reductions in innate pulmonary immune responses and the development of pneumonia in IAV-infected animals. Taken together, these data indicate a critical role for pneumococcal metabolism in shaping host responses and altering disease severity during postinfluenza bacterial pneumonia.

## RESULTS

### Bacterial adaptations during pneumococcus coinfection with influenza virus.

**FIG 1:**
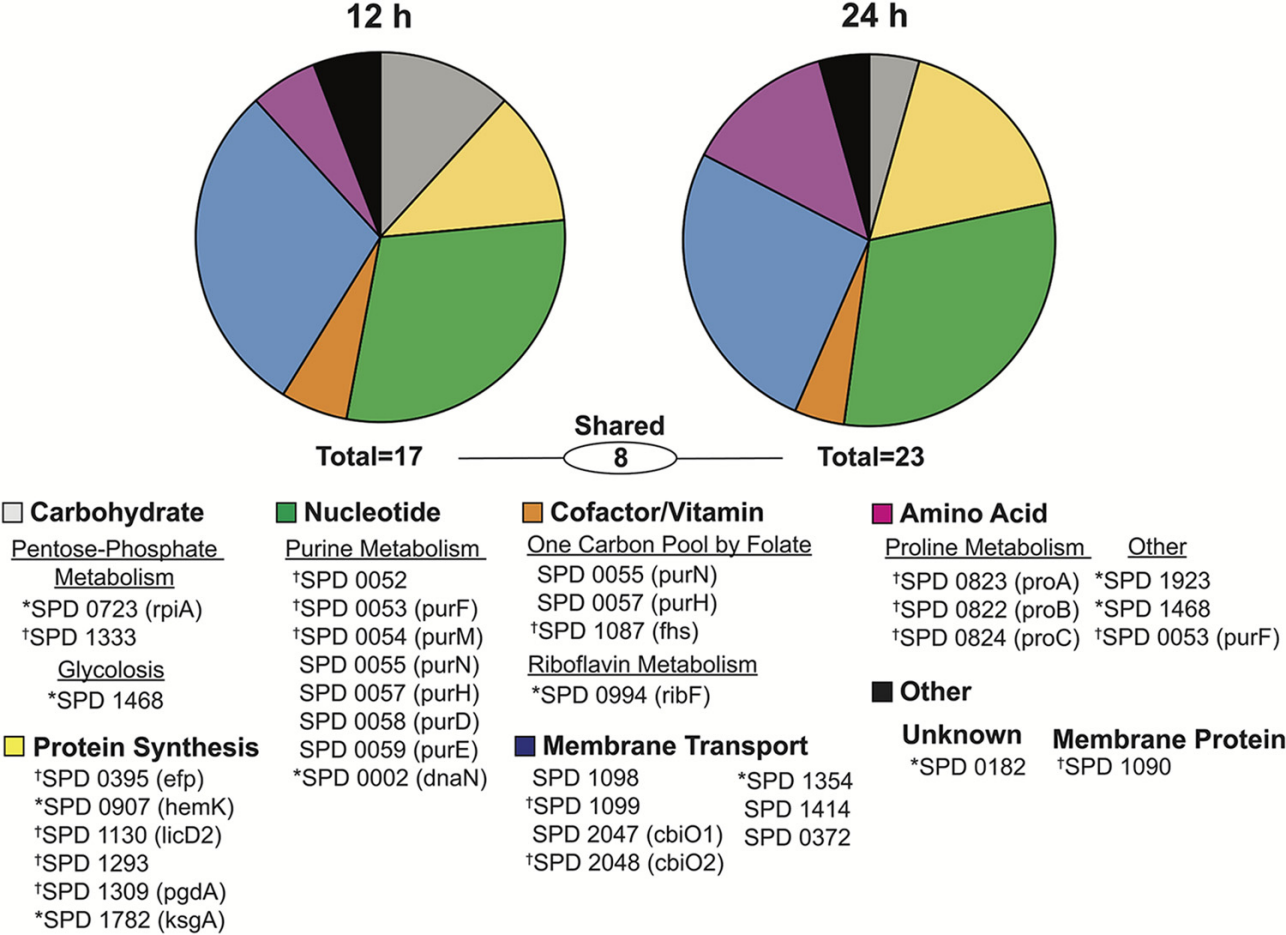
Time-dependent analysis of pneumococcal genes impacting fitness during influenza virus coinfection. Functional breakdown of the pneumococcal genes that impact bacterial fitness during coinfection with influenza A virus as identified by Tn-Seq. Markers identify genes important only at 12 h pbi* (17 total) or only at 24 h pbi^†^ (23 total). The absence of a marker indicates significance at both time points (8 total).

To identify the pneumococcal factors that increase bacterial fitness during IAV infection, we employed a high-throughput transposon sequencing (Tn-Seq) approach (52). We generated a library of ∼50,000 transposon insertion mutants in the type 2 pneumococcal strain D39 and then infected groups of mice with 75 50% tissue culture infectious doses (TCID_50_) of influenza virus A/Puerto Rico/34/8 (PR8) or the mock control (phosphate-buffered saline [PBS]), followed 7 days later with 10^6^ CFU of the transposon library. Bacteria were collected from lungs harvested at 12 h or 24 h post-bacterial infection (pbi), genomic DNA was isolated and sequenced, and the relative abundances and fitness of transposon mutants were calculated (see the supplemental material). Bacterial fitness was first compared pre- and postinfection and then compared between mock- and IAV-infected animals, to identify the differences in genes required for fitness. This analysis highlighted 17 genes at 12 h pbi and 23 genes at 24 h pbi that were required for fitness in IAV-infected hosts (Table 1 and Fig. 1). Of these, 8 genes were detected at both time points. The core set of genes identified at 12 h and 24 h pbi are responsible for amino acid biosynthesis, nucleotide biosynthesis, protein translation, and membrane transport (Fig. 1). Genes in the purine biosynthesis pathway comprised the largest number of genes (8 total: SPD0002 [*dnaN*], SPD0052, SPD0053 [*purF*], SPD0054 [*purM*], SPD0055 [*purN*], SPD0057 [*purH*], SPD0058 [*purD*], and SPD0059 [*purE*]), followed by ATP-binding cassette (ABC) transporters (5 total: SPD1098, SPD1099, SPD2047 [*cbiO1*], SPD2048 [*cbiO2*], and SPD1354 [putative]), protein translation (6 total: SPD0395 [*efp*], SPD1782 [*ksgA*], SPD0907 [*hemK*], SPD1130 [*licD2*], SPD1293, and SPD1923), and proline biosynthesis (3 total: SPD0822 [*proB*], SPD0823 [*proA*], and SPD0824 [*proC*]). Other genes included a putative membrane protein (SPD1090), carbon metabolism (SPD0723 [*ripA*], SPD1087, SPD1333 [putative], and SPD1468), and riboflavin metabolism (SPD0994).

**TABLE 1:** Pneumococcal genes with significant differential fitness during coinfection with IAV^*a*^

Locus	Gene	Description	Identified at indicated time point (h pbi)	
			12	24
SPD_0002	*dnaN*	DNA polymerase III subunit beta	×	
SPD_0052		Phosphoribosylformylglycinamidine synthase, putative		×
SPD_0053	*purF*	Amidophosphoribosyltransferase		×
SPD_0054	*purM*	Phosphoribosylformylglycinamidine cyclo-ligase		×
SPD_0055	*purN*	Phosphoribosylglycinamide formyltransferase	×	×
SPD_0057	*purH*	Bifunctional purine biosynthesis protein PurH	×	×
**SPD_0058**	***purD***	**Phosphoribosylamine-glycine ligase**	×	×
SPD_0059	*purE*	Phosphoribosylaminoimidazole carboxylase, catalytic subunit	×	×
SPD_0182		Conserved hypothetical protein	×	
SPD_0372		Sodium:alanine symporter	×	×
SPD_0395	*efp*	Translation elongation factor P		×
SPD_0723	*rpiA*	Ribose 5-phosphate isomerase A	×	
**SPD_0822**	***proB***	**Glutamate 5-kinase**		×
SPD_0823	*proA*	Gamma-glutamyl phosphate reductase		×
SPD_0824	*proC*	Pyrroline-5-carboxylate reductase		×
SPD_0907	*hemK*	HemK protein	×	
SPD_0994	*ribF*	Riboflavin biosynthesis protein RibF	×	
SPD_1087	*fhs*	Formate-tetrahydrofolate ligase		×
SPD_1090		Membrane protein, putative		×
**SPD_1098**		**Amino acid ABC transporter, amino acid-binding protein/permease protein**	×	×
SPD_1099		Amino acid ABC transporter, ATP-binding protein		×
SPD_1130	*licD2*	Phosphotransferase LicD2		×
SPD_1293		Acetyltransferase, GNAT family protein		×
SPD_1309	*pgdA*	Peptidoglycan GlcNAc deacetylase		×
SPD_1333		Conserved hypothetical protein		×
SPD_1354		Conserved hypothetical protein	×	
**SPD_1414**		**Oxalate:formate antiporter**	×	×
SPD_1468		Phosphoglycerate mutase	×	
SPD_1782	*ksgA*	Dimethyladenosine transferase	×	
SPD_1923		2,3,4,5-Tetrahydropyridine-2-carboxylate *N*-succinyltransferase, putative	×	
**SPD_2047**	***cbiO1***	**Cobalt ABC transporter, ATP-binding protein CbiO1**	×	×
SPD_2048	*cbiO2*	Cobalt ABC transporter, ATP-binding protein CbiO2		×

Total			17	23

*^a^*Pneumococcal fitness genes identified at 12 h and 24 h pbi by Tn-Seq during IAV infection compared to mock-infected hosts. Highlighted in boldface are the genes chosen for additional characterization.

### Impaired bacterial metabolism selectively reduces fitness *in vitro*.

**FIG 2:**
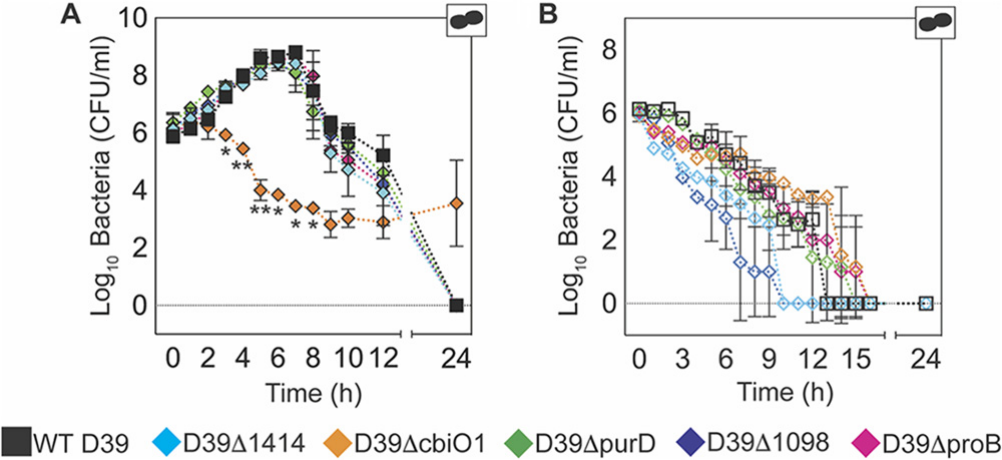
*In vitro* growth kinetics of SGD mutant bacteria. Bacteria were grown at 37°C in 1 ml of THY medium (A) or PBS (B). *, *P* < 0.05, and **, *P* < 0.01 (D39Δ*cbiO1* only), for significance compared to WT D39 at the indicated time point (A). Each symbol (squares or diamonds) shows the mean value from two representative experiments, and the bars show the geometric mean values ± standard deviations (SD). Cartoons indicate pathogens in the culture (bacteria alone).

We generated single-gene-deletion (SGD) bacterial mutants from 3 of the categories identified (D39Δ*cbiO1*, D39Δ*1414*, D39Δ*1098* [membrane transport], D39Δ*purD* [purine metabolism], and D39Δ*proB* [proline metabolism]) to assess the differential fitness conveyed by genes predicted by Tn-seq (Table 1, Fig. 1, and Table S1). The growth of the SGD mutants in culture medium was unaffected, with the exception of D39Δ*cbiO1*, which was significantly attenuated from 3 h to 8 h (*P* < 0.05) and was the only SGD strain that had not autolysed after 24 h (Fig. 2A). During metabolic starvation (i.e., in PBS), D39Δ*1098* decayed more rapidly (*P* = 0.01) and D39Δ*cbiO1* more slowly (*P* < 0.01), but other SGD mutants were similar to the wild type (WT) (Fig. 2B). Bacterial growth was rescued when PBS cultures were supplemented with lung homogenate supernatants (s/n) (Fig. S1A and B in the supplemental material) and was not different than that of the WT strain D39 after 6 h of culture in lung homogenate s/n from mock- or IAV-infected mice (75 TCID_50_ PR8, 7 days) (Fig. S1C and D).

### Impaired bacterial metabolism protects against virulence *in vivo.*

**FIG 3:**
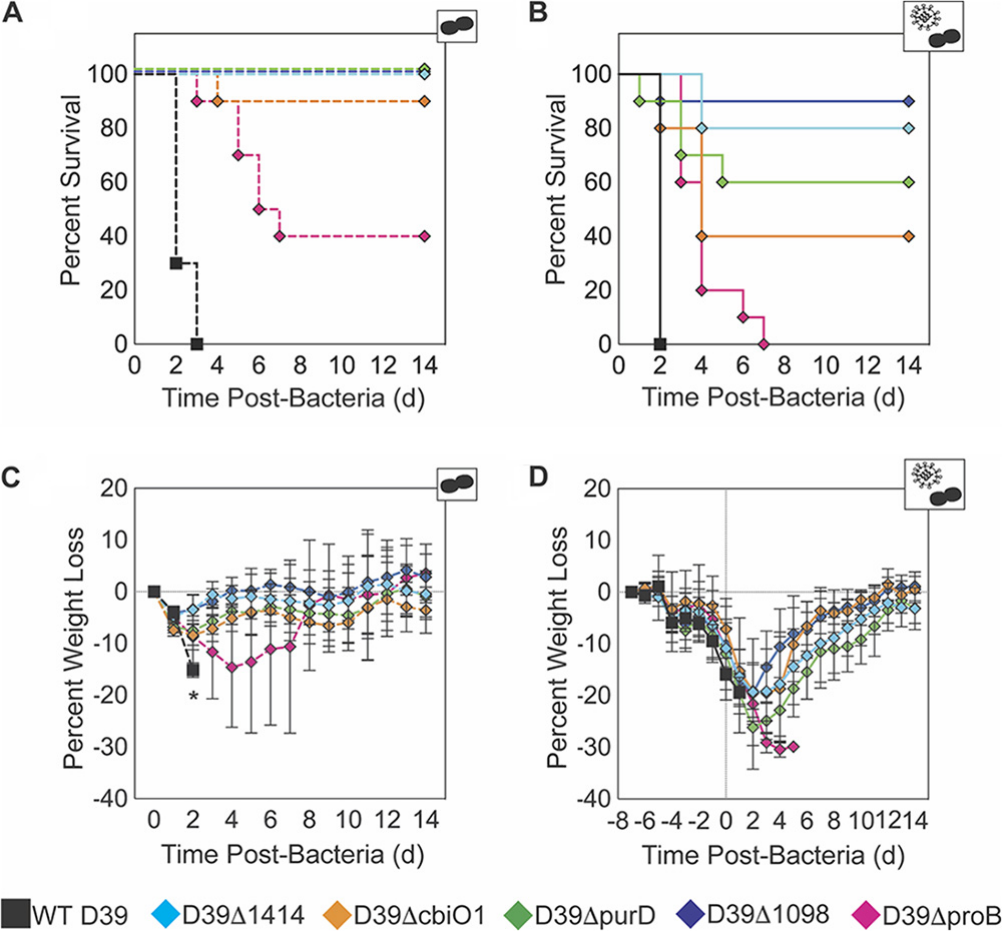
*In vivo* pathogenicity of infection with SGD mutant bacteria. Kaplan-Meier survival curves (A, B) and weight loss (percent loss compared to naive mice) (C, D) of mice that were mock infected (PBS) (dashed lines; A and C) or IAV infected (75 TCID_50_ PR8) (solid lines; B and D), followed 7 days later with 10^6^ CFU of the indicated bacteria. Survival curves are significantly different (*P* < 0.01) for each SGD mutant compared to the results for WT D39 in mock-infected (A) and IAV-infected (B) animals. Differences in survival curves are detailed in Table 2. *, *P* < 0.05, for significant difference in weight loss with each SGD mutant (diamonds) compared to WT D39 (square) at the indicated time point (C). Data are shown as the mean values ± standard deviations (SD) from 10 mice/group. Cartoons indicate infection status of study group (bacteria alone or virus plus bacteria).

**(i) Mortality is reduced.** To establish the effect of gene deletion on pathogenicity, weight loss was monitored as a measure of disease severity (Fig. 3 and Table 2). In mock- and IAV-infected animals, infection with WT D39 resulted in 100% mortality by 72 h pbi and 48 h pbi, respectively (Fig. 3A and B). In mock-infected animals, mortality was reduced by 90 to 100% with 4 of 5 SGD mutants and by 40% with D39Δ*proB* (all *P* < 0.01) (Fig. 3A and Table 2). Correspondingly, weight loss was significantly reduced (48 h pbi; *P* < 0.05) (Fig. 3C). In IAV-infected animals, mortality was reduced by 40 to 90% with 4 out of 5 SGD mutants (all *P* < 0.01) (Fig. 3B and Table 2). Coinfection with D39Δ*proB* resulted in 100% mortality; however, the mean survival time was lengthened by 5 days (*P* < 0.01) (Fig. 3B and Table 2). In IAV-infected animals, weight loss was not significantly reduced compared to that of WT D39-infected animals (24 h pbi; *P* > 0.05) (Fig. 3D), and the weights of all SGD-infected animals, except those infected with D39Δ*proB*, began to rebound ∼3 days pbi and returned to baseline by 12 to 14 days pbi.

**TABLE 2:** Summary of pathogenicity of SGD mutant bacteria with or without influenza A virus strain PR8 coinfection

Bacterial strain	Log_10_ change in^*a*^:										% survival of mice with indicated treatment^*b*^	
	Lung bacteria				Blood bacteria				Virus (PR8 + bacteria)			
	PBS + bacteria		PR8 + bacteria		PBS + bacteria		PR8 + bacteria				PBS	PR8
	4 h	24 h	4 h	24 h	4 h	24 h	4 h	24 h	4 h	24 h		
D39Δ*1414*	−0.61****	−2.71	−0.94****	−0.83**	−3.79	−5.87	−1.65	−4.97**	0.02	0.43	100****	80****
D39Δ*cbiO1*	−1.36****	−5.86	−2.05****	−2.11**	−2.31	−6.56	−1.38	−2.35**	0.15	0.29	90****	40***
D39Δ*purD*	−0.74****	−0.47	−0.44****	−0.87*	−1.49	−1.28	−0.40	−2.64**	−0.83	0.26	100****	60***
D39Δ*1098*	−0.59****	−2.17	−0.76****	−0.78**	−3.78	−3.51	−1.69	−2.25**	−0.83	−0.93	100****	90****
D39Δ*proB*	−1.07****	−1.70	−1.08****	−0.86*	−3.21	−6.56	−1.86	−2.98**	−0.04	−0.19	40****	0****^c^

*^a^*Log_10_ changes in the levels of lung bacteria, blood bacteria, and lung virus from mice (groups of 5) that were either mock infected (PBS) or IAV infected (75 TCID_50_ PR8), followed 7 days later by 10^6^ CFU of the indicated bacteria. Comparisons were made between average log_10_ bacteria or virus at 4 h pbi and 24 h pbi. Significance in analysis of variance (ANOVA) using a Dunnett correction for multiple comparisons (to WT D39) is indicated by boldface.

*^b^*Percent increases in survival of animals (groups of 10) that were either mock infected (PBS) or IAV infected (75 TCID_50_ PR8), followed 7 days later by 10^6^ CFU of the indicated bacteria. Significant differences in Kaplan-Meier survival curves by the log rank test are indicated by boldface. *, *P* < 0.05; **, *P* < 0.01; ***, *P* < 0.005; ****, *P* < 0.0001.

*^c^*Mean survival time at 7 days pbi.

**FIG 4:**
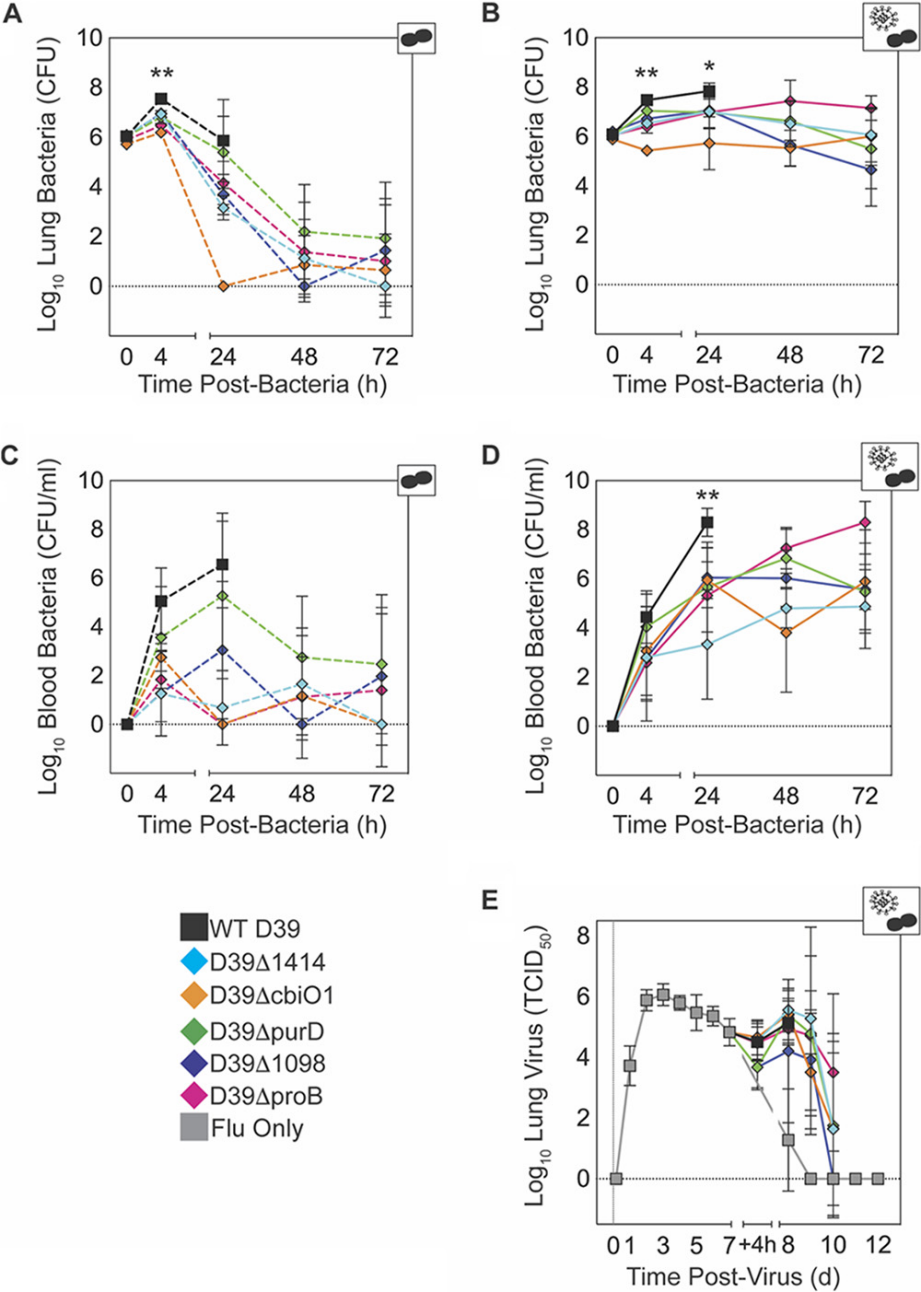
Viral and bacterial titer kinetics. Lung bacterial titers (A, B), blood bacterial titers (C, D), and lung viral titers (E) from groups of mice either mock infected (PBS) (dashed lines in A and C) or IAV infected (75 TCID_50_ PR8) (solid lines in B, D, and E), followed 7 days later with 10^6^ CFU of the indicated bacteria. *, *P* < 0.05 and **, *P* < 0.01, for significance for each of the SGD mutants compared to WT D39 at the indicated time point. Data are shown as the mean values ± standard deviations (SD) from 5 mice/group. Cartoons indicate infection status of study group (bacteria alone or virus plus bacteria). The log_10_ changes in pathogen loads between each SGD mutant and WT D39 and analysis of variance results are summarized in Table 2.

**(ii) Bacteria in the lung and blood are reduced.** The lung titer kinetics of the SGD mutants *in vivo* (Fig. 4A and B) mirrored the *in vitro* titer kinetics in lung s/n-supplemented cultures (Fig. S2C and D). At 4 h pbi, SGD mutant lung titers were 0.6 to 1.4 log_10_ CFU lower than those of WT D39 in mock-infected animals and 0.4 to 2.1 log_10_ CFU lower in IAV-infected animals (*P* < 0.01) (Fig. 4A and B and Table 2). By 24 h pbi, titers were statistically similar and yet reduced compared to those of WT D39 in the lungs (0.5 to 2.7 log_10_ CFU reduction; *P* > 0.05) and blood (1.3 to 6.6 log_10_ CFU reduction; *P* > 0.05) of mock-infected animals (Fig. 4A and C and Table 2). However, in IAV-infected animals, SGD mutant titers remained significantly lower in the lungs (0.8 to 2.1 log_10_ CFU reduction; *P* < 0.05) and blood (2.2 to 5.0 log_10_ CFU reduction; *P* < 0.01) at 24 h pbi (Fig. 4B and D and Table 2). Despite attenuated growth in the lungs and reduced bacteremia at 24 h pbi, the bacterial loads for each SGD mutant remained high in the lungs and blood of coinfected animals at 24 h, 48 h, and 72 h pbi (Fig. 4B and D and Table 2).

**(iii) IAV kinetics are similar.** Similar to previous studies (53), viral loads rebounded following coinfection with each SGD mutant bacterial strain. While viral rebound and clearance occurred with various dynamics, each was statistically similar to those found during coinfection with WT D39 (*P* > 0.05) (Fig. 4E and Table 2).

**FIG 5:**
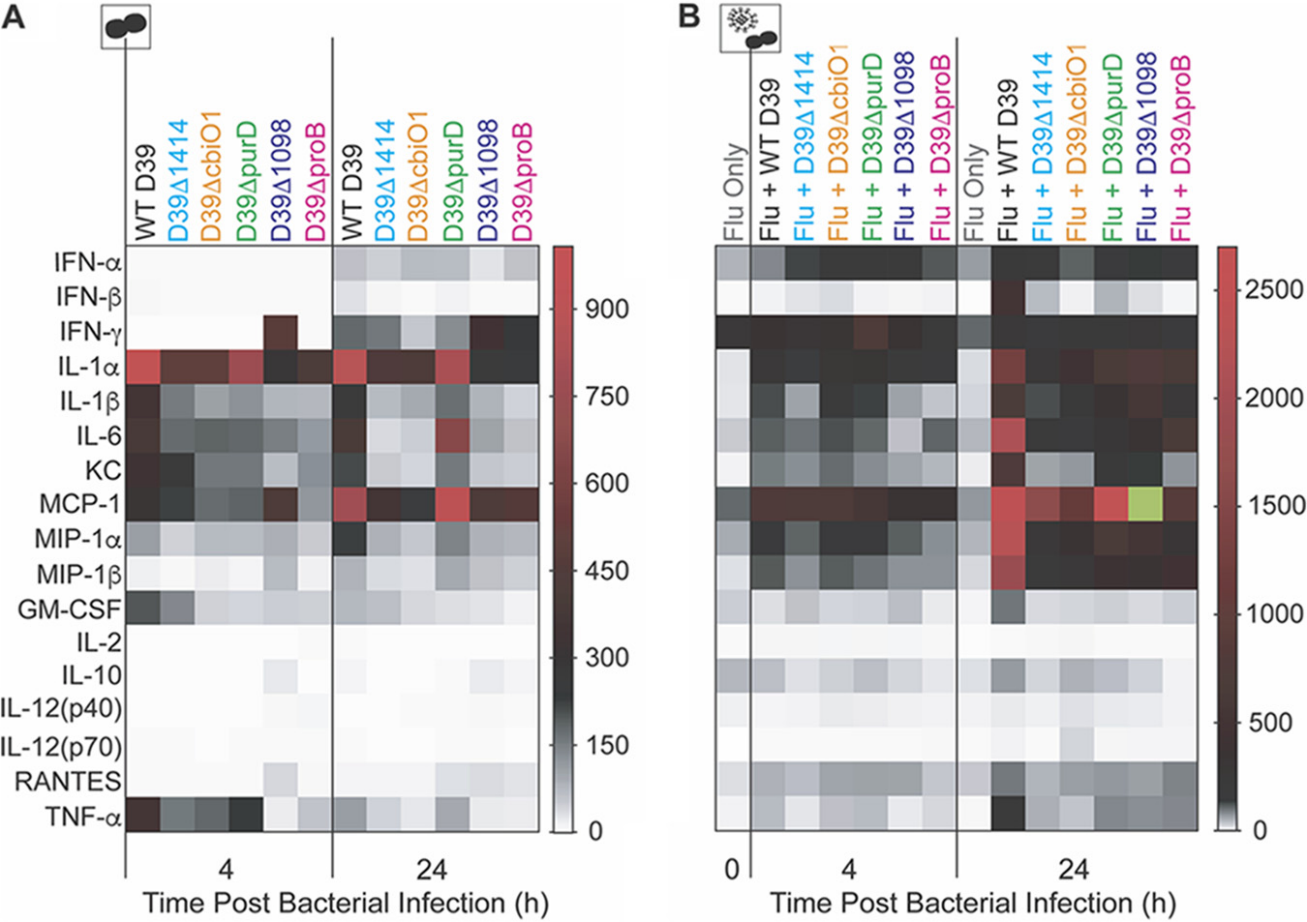
Heat map of cytokine and chemokine changes. Fold change compared to naive in the mean value of the indicated cytokine/chemokine at 4 h or 24 h pbi in the lungs of mice that were mock infected (PBS) (A) or IAV infected (75 TCID_50_ PR8) (B), followed 7 days later with 10^6^ CFU of the indicated bacteria. The green square is outside the listed range and indicates a 4,021-fold change. Cartoons indicate infection status of study group (bacteria alone or virus plus bacteria). Plots depicting absolute log_10_ picograms (pg) of measured cytokines and chemokines are in Fig. S2 to S4 in the supplemental material.

**(iv) Select cytokine and chemokine responses are altered.** To better understand the reduced morbidity and mortality during infection with the SGD mutants, we examined cytokine and chemokine dynamics in the lungs (Fig. 5 and Fig. S2 to S4). In IAV-infected animals, interferon alpha (IFN-α) and IFN-β were significantly reduced at 24 h pbi during infection with SGD mutant bacteria compared to their levels during infection with WT D39 (*P* < 0.05), with the exception of IFN-α during coinfection with D39Δ*1414* (*P* = 0.95) and D39Δ*purD* (*P* = 0.17) (Fig. 5 and Fig. S2J and L). Interestingly, at 4 h pbi in IAV-infected animals, IFN-α was elevated in D39Δ*1414*, D39Δ*cbiO1*, D39Δ*purD*, and D39Δ*1098* coinfections (*P* < 0.01) and IFN-β was elevated in D39Δ*cbiO1* coinfection (*P* < 0.01) (Fig. 5 and Fig. S2J and L). In mock-infected animals, IFN-α was unaltered (*P* > 0.05) and IFN-β was reduced at 24 h pbi during infection with only D39Δ*cbiO1*, D39Δ*1098*, and D39Δ*proB* (*P* < 0.05) (Fig. 5 and Fig. S2I and K). At 24 h pbi, interleukin-6 (IL-6), KC, MIP-1β, and granulocyte-macrophage colony-stimulating factor (GM-CSF) were reduced during infection with all SGD mutant bacteria in IAV-infected animals (*P* < 0.05), while only GM-CSF was reduced with D39Δ*1098* (*P* < 0.05) and D39Δ*proB* (*P* < 0.01) in mock-infected animals (Fig. 5 and Fig. S2 to S4). IL-1α, IL-1β, MIP-1α, and tumor necrosis factor alpha (TNF-α) were reduced in both IAV- and mock-infected animals at 24 h pbi during infection with each SGD mutant compared to their levels during infection with WT D39 (*P* < 0.05), except for D39Δ*purD* in mock-infected animals (*P* > 0.05) (Fig. 5 and Fig. S2 to S4). Minimal differences were detected for monocyte chemoattractant protein 1 (MCP-1), RANTES, IL-2, IFN-γ, IL-10, IL-12(p40), and IL-12(p70), except during primary infection with D39Δ*1098* and D39Δ*proB* (Fig. 5 and Fig. S3 and S4). Overall, there were reductions in inflammatory cytokines and chemokines during infection with SGD mutant bacteria compared to their levels during infection with WT D39, and the extent and timing of these reductions differed based on the IAV infection status of the host.

**FIG 6:**
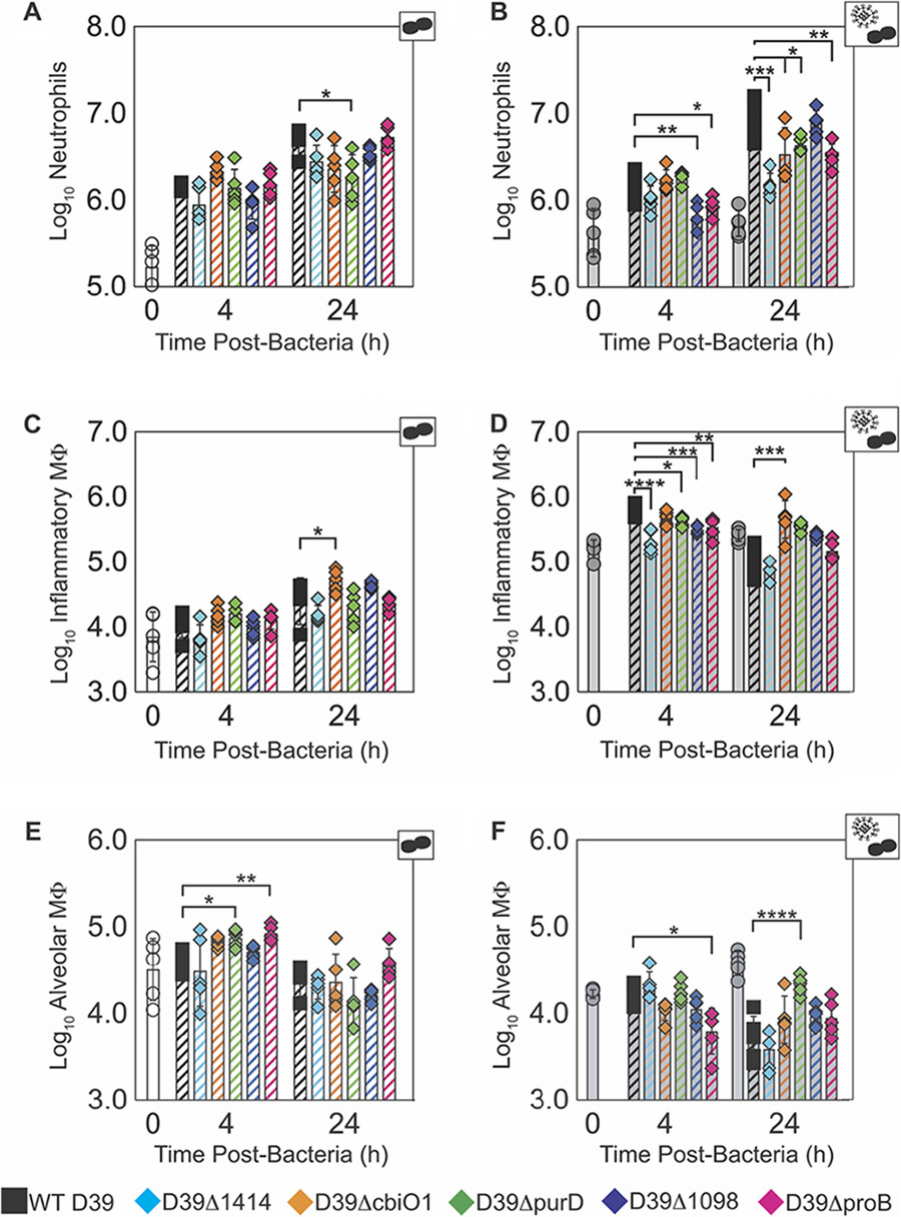
Pulmonary immune cell kinetics. Kinetics at 4 h or 24 h pbi of neutrophils (A, B), inflammatory macrophages (C, D), and alveolar macrophages (E, F) from mice that were mock infected (PBS) (A, C, E) or IAV infected (75 TCID_50_ PR8) (B, D, F), followed 7 days later with 10^6^ CFU of the indicated bacteria. Each symbol (circles, squares, or diamonds) represents the value for a single mouse, and the bars show the geometric mean values ± standard deviations (SD) from 5 mice/group. Mice were either uninfected (open [white]), infected with IAV only for 7 days (*t* = 0) or 8 days (*t* = 24) (solid [light gray]), infected with bacteria (open, hatched, colored), or coinfected with IAV and bacteria (solid, hatched, colored). *, *P* < 0.05; **, *P* < 0.01; ***, *P* < 0.005; ****, *P* < 0.0001. Cartoons indicate infection status of study group (bacteria alone or virus plus bacteria). The flow cytometry gating scheme is in Fig. S5, and additional cellular dynamics are in Fig. S6.

**(v) Inflammatory cell responses are reduced.** In accordance with the changes detected in pulmonary cytokines and chemokines, infection with SGD mutant bacteria altered the dynamics of select immune cells in the lungs (Fig. 6 and Fig. S5-S6). In mock-infected animals, neutrophils (Ly6G^hi^) were not different at 4 h pbi during infection with any of the SGD mutants (*P* > 0.05) and were only reduced at 24 h pbi by D39Δ*purD* (*P* < 0.05) (Fig. 6A). However, in IAV-infected animals, neutrophils were reduced at 4 h pbi with D39Δ*1098* (*P* < 0.01) and D39Δ*proB* (*P* < 0.05) and at 24 h pbi with D39Δ*1414*, D39Δ*cbiO1*, D39Δ*purD*, and D39Δ*proB* (*P* < 0.05) (Fig. 6B). Inflammatory macrophages (IMΦ; Ly6G^−^, CD11c^hi^, F4/80^hi^, CD11b^+^) were also similar at 4 h pbi for all of the SGD mutants in mock-infected animals (*P* > 0.05) and were increased by only D39Δ*cbiO1* infection at 24 h pbi (*P* < 0.05) (Fig. 6C). However, in IAV-infected animals, IMΦ were reduced at 4 h pbi during infection with D39Δ*1414*, D39Δ*purD*, D39Δ*1098*, and D39Δ*proB* (*P* < 0.05) (Fig. 6D). D39Δ*cbiO1* did not lead to reduced IMΦ at 4 h pbi (*P* = 0.14) but did induce an increase in IMΦ at 24 h pbi (*P* < 0.01) that was not observed with the other SGD mutants (Fig. 6D). There were minimal differences in T cell populations (Fig. S6I to L) or the extent of AMΦ depletion (Ly6G^−^, CD11c^hi^, F4/80^hi^, CD11b^−^) (Fig. 6E and F) in mock- or IAV-infected animals. Thus, infection with SGD mutant bacteria induced less neutrophil and IMΦ infiltration in a time-dependent manner in IAV-infected hosts, but these cells were largely unaltered in mock-infected hosts.

**FIG 7:**
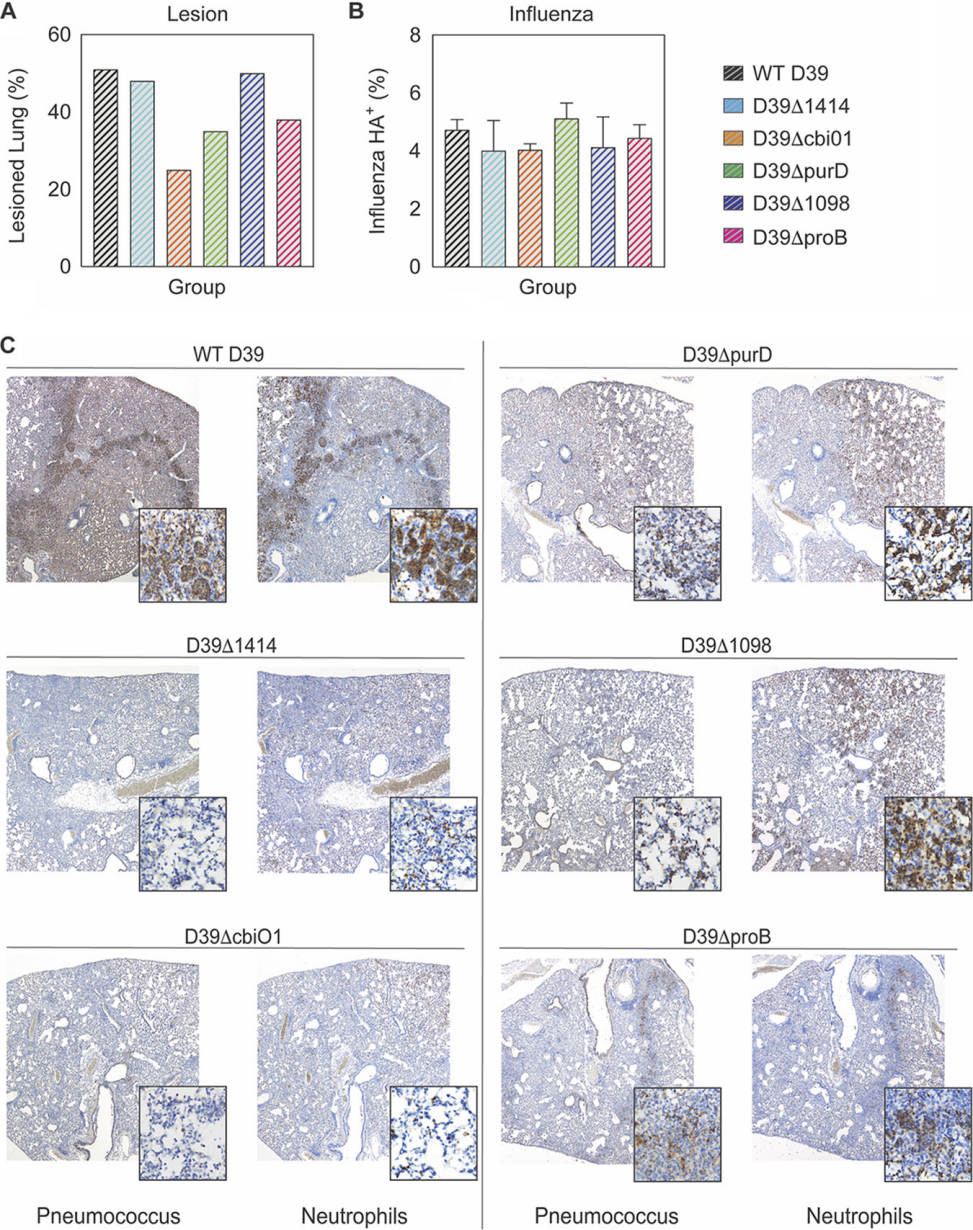
Lung pathology during IAV-pneumococcus coinfection. Serial sections of lungs at 24 h pbi from mice that were IAV infected (75 TCID_50_ PR8), followed 7 days later with 10^6^ CFU of the indicated bacteria, were stained with hematoxylin and eosin (HE) for histological analysis (A) or immunohistochemistry (IHC) for influenza virus HA glycoprotein (B), pneumococcus, or neutrophil Ly6G/6C antigen (C). Quantification was performed by a pathologist blinded to the study design (A) or with MIPAR version 3.0 image analysis software (B) (Fig. S7). Bar graphs depict the percent area positive in mice used for representative images (A) or the geometric mean ± standard deviation of sections from 2 mice/group (B). Representative images are at an original ×4 magnification with ×60 magnification insets.

**(vi) Pathology and neutrophil degeneration are reduced.** There was extensive pulmonary consolidation at 24 h pbi in the lungs of mice coinfected with WT D39, as characterized by thickened septa and alveoli filled with a mixture of neutrophils, free bacteria, and proteinaceous exudates (Fig. 7A). These lesions were dramatically reduced during coinfection with D39Δ*proB*, D39Δ*1098*, *and* D39Δ1414 and almost absent in D39Δ*cbiO1* and D39Δ*purD* coinfection (Fig. 7A). Immunohistochemical (IHC) staining showed a similar extent of influenza virus antigen (Fig. 7B) but dramatically less bacterial antigen at 24 h pbi during coinfection with each of the SGD mutants (Fig. 7C and Fig. S7A), which mirrored the pulmonary viral and bacterial loads (Fig. 4B and E). In WT D39-coinfected animals, intracellular and extracellular bacterial antigen was present throughout areas showing influenza lesions, including perivascular connective tissues, consolidated alveolar parenchyma, and the central hypocellular area of resolving lesions. Bacterial antigen was not detected in the resolving influenza lesions with any of the SGD mutants, except for D39Δ*1098*, where few bacteria or pneumococcal-antigen-positive macrophages were present. IHC staining also showed massive neutrophil infiltration in WT D39 coinfection (Fig. 7C), and the resolving influenza lesions were consistently surrounded by sharply demarcated hypercellular bands composed of viable neutrophils and those undergoing hydropic degeneration as noted by a board-certified pathologist. Degenerating neutrophils histologically presented with swollen smudged nuclei undergoing lysis and were often admixed with fibrin, edema fluid, and necrotic cell debris. In contrast, during D39Δ*proB*, D39Δ*purD*, and D39Δ*1098* coinfections, neutrophilic infiltrates generally appeared in clusters within alveoli peripheral to influenza lesions or scattered widely throughout inflamed areas in the lungs. Neutrophil infiltrates in these lungs consisted mostly of intact, nondegenerating cells that formed indistinct bands surrounding the influenza lesions (Fig. 7C). Animals coinfected with D39Δ*1414* and D39Δ*cbiO1* had the lowest numbers of neutrophils, which were mostly nondegenerating and scattered throughout inflamed areas (Fig. 7C). Neutrophils were rare or absent within the resolving influenza lesions in animals coinfected with an SGD mutant.

## DISCUSSION

Pathogenicity during IAV-pneumococcus coinfection is influenced by several pathogen and host factors, including the viral and bacterial strains and doses and the strength of the inflammatory response (11–16). Our study presented here highlights the importance of specific genetic factors in contributing to this pathogenicity and to the dysregulated host response. By using Tn-Seq as an unbiased approach, we identified 32 genes as being critical to pneumococcal survival in the IAV-infected host in a time-dependent manner (Table 1 and Fig. 1). Unsurprisingly, known bacterial virulence genes were not varied in our screen, likely because those factors affect disease equally regardless of viral infection status (40, 41, 43). The genes identified are mostly involved in bacterial metabolism, which supports findings that the lung metabolome is altered during influenza virus infection. Moreover, this selective pressure of the host environment suggests that equivalent bacterial alterations may occur across coinfecting pneumococcal strains and serotypes, but only the D39 strain is assessed herein. Our *in vivo* data suggest that some identified genes function as virulence factors in both mock- and IAV-infected hosts. However, the immunomodulatory effects differed dramatically between heathy mice and those infected with the influenza virus, highlighting the importance of immune and metabolic alterations in the susceptibility to and severity of bacterial coinfection.

Altered metabolic regulation has been observed during murine influenza virus infection (54–57) and *in vitro* influenza virus infection of primary human bronchotracheal epithelial (HBAE) cells (58, 59) and in pediatric patients infected with influenza virus (58). However, even with the knowledge that influenza virus infection induces metabolic changes and that pneumococci modulate metabolic pathways under host-specific pressures (51, 60), the collection of genes identified here was not intuitive, nor was their precise contribution to the infection dynamics. It has been shown that purine metabolic pathways are altered in Haemophilus influenzae and pneumococcus (D39 strain) within influenza virus-infected hosts (49, 50) and in pneumococcus (TIGR4 strain) within hosts with sickle cell disease (51). Here, eight genes with roles in purine metabolism were identified, including SPD0058 (*purD*) (Table 1 and Fig. 1) (61–63). This could indicate a common mechanism for bacterial adaptation within inflammatory environments. In influenza virus-infected hosts, purine biosynthesis is upregulated (55) and purine analogs can be used to reduce disease severity (e.g., by the antiviral T-705 [64, 65]). However, the intermediates of purine biosynthesis become depleted following bacterial infection (50), rendering genes of *de novo* purine intermediate synthesis (*purD* and *purC*) critical to coinfecting bacteria (Table 1 and Fig. 1) (50, 51). Here, it is likely that changes in purine scavenging and competition for environmentally available purines/purine intermediates modulate host immune cell function during infection with D39Δ*purD*, contributing to decreased lethality (Fig. 3B) and immunopathology (Fig. 7A) in IAV-infected hosts.

Several of the genes identified here act in glutamate/glutamine biosynthesis, which, while important for bacterial pathogenesis in otherwise healthy individuals, plays a key role during influenza virus-pneumococcus coinfection. Here, both genes in the locus pinpointed as the main ABC glutamine/glutamate transporter of pneumococci (SPD1098/1099) (66, 67) were identified in our screen (Table 1 and Fig. 1). Deleting SPD1098 increased survival by 90% in coinfected animals (Fig. 3B), despite neutrophil accumulation equivalent to that in WT D39 coinfection (Fig. 6B and Fig. 7C). Genes involved in proline biosynthesis, a process downstream from glutamate metabolism (61–63), were also identified, including SPD0822/SPD0823 (*proB*/*proA*) (Table 1 and Fig. 1). Deletion of SPD0822 (*proB*) delayed mortality during coinfection (Fig. 3B) and led to reduced neutrophils and IMΦ in influenza virus-infected hosts, but not in mock-infected hosts (Fig. 6). Glutamine is utilized at a high rate by immune cells and is needed for optimal function of macrophages and neutrophils (68–73). Since pulmonary cells have increased dependence on glutamine during influenza virus infection (57, 58), it is probable that competition for limited environmentally available glutamine altered neutrophil infiltration and function, thereby reducing morbidity and mortality in IAV-infected hosts coinfected with these SGD mutants (Fig. 3B and 5 to 7). Investigating metabolic alterations of innate immune cells during IAV infection would clarify the host-pathogen interactions observed here.

The functions of some genes identified here, including three other ABC transporters (e.g., SPD2047/SPD2048 [*cbiO1*/*cbiO2*] and SPD1414), are not well characterized. While it is understood that ABC transporters are important for pneumococcal virulence (35, 36, 74–77), investigating the impact of each gene identified in our screen provides insight into respiratory changes that occur during influenza virus infection. The role of cobalt in pneumococcal physiology and virulence remains unclear (78, 79), but the *cbiO* locus (putative cobalt transporter) was previously identified by microarray analysis as necessary for pneumococcal pathogenicity (42). Interestingly, bacteria lacking SPD2047 reach significantly higher lung titers in IAV-infected animals than in mock-infected animals (Fig. 4A to D), suggesting that cobalt metabolism may be modified by influenza virus infection. To our knowledge, no study has assessed cobalt dynamics during influenza. The TIGR4 analog of the oxalate/formate antiporter SPD1414 (SP1587) has been shown to be important for bacterial survival in the lungs and blood (36, 80), cerebrospinal fluid (36), and nasopharynx (80). Here, deletion of SPD1414 reduced lethality (Fig. 3B), neutrophil infiltration, and pulmonary damage in IAV-infected hosts (Fig. 6 and 7). These results indicate roles for cobalt and oxalate/formate in the susceptibility to pneumococcal pneumonia during influenza virus infection.

Mortality during influenza virus-pneumococcus coinfection is typically associated with an exuberant immune response coupled with high pathogen loads in the lung and the blood (14–16, 24, 81–83). However, reduced inflammation can lessen disease severity even with sustained bacterial loads (26). Here, the growth of SGD mutant bacteria in IAV-infected animals was significantly attenuated (0.8 to 2.1 log_10_ reduction compared to the growth of WT D39) but not strictly correlated with pathogenicity and insufficient to account for the extreme reductions in mortality (up to 90%) (Fig. 3B and 4 and Table 2), as equivalently reduced bacterial loads of WT D39 in IAV-infected animals still induce significant mortality (Fig. S8). In addition, AMΦ, which dictate the initial pneumococcal invasion and growth kinetics during IAV infection (18–20, 23), are not different during coinfection with most SGD mutants than with WT D39 (Fig. 6F). The contractions of the pulmonary CD8^+^ T cells (Fig. S6L) (84) and the rebounds of viral loads (Fig. 4E) were not different during coinfection with SGD mutant bacteria, which supports our previous findings that the mechanisms underlying rapid bacterial growth are independent from those that influence the postbacterial infection viral rebound and pathogenicity (19, 20). These findings suggest that the observed improvements in immune responses and disease outcome are not solely driven by the differences in bacterial loads. Reduced cytokine levels, specifically, lowered type I IFNs (Fig. 5 and Fig. S2J and L), can improve neutrophil function (21, 24, 25, 85–89) and reduce epithelial cell death and lung permeability (90) and, thus, reduce pathogenicity (Fig. 3B, 5, and 7). Moreover, reduced neutrophil infiltration and degeneration (Fig. 6B and 7C) may have mitigated the damaging cytokine storm that is typically associated with IAV-pneumococcus pneumonia (12–17, 33). A direct correlation between survival and any single host immune response in the early stages of coinfection (0 to 24 h pbi) was not readily apparent. It is possible that unmeasured components and/or cumulative effects influence lethality at later time points. These studies underscore the independent nature of pathogen growth and pathogenicity and illuminate the difficulty in reducing coinfection pathogenicity to a single variable.

Understanding how bacterial adaptations influence the development of pneumonia during influenza virus infections is important to effectively combat the disease. Here, we provide insight into the contribution of specific pneumococcal genes and into the regulatory host-pathogen dynamics that arise during IAV-pneumococcus coinfection. Our findings highlight the critical role of influenza virus-induced metabolic shifts in promoting bacterial infection and altering immune function and suggest that targeting a single pneumococcal gene or metabolite could be an effective intervention to abrogate bacterial pneumonia during influenza virus infection. Further dissecting bacterial adaptations in other pneumococcal serotypes and strains and coinfecting species may help identify therapeutic targets that could be used to prevent or treat postinfluenza bacterial infections.

## MATERIALS AND METHODS

### Ethics statement.

All experimental procedures were performed under protocol O2A-020 approved by the Animal Care and Use Committee at St. Jude Children’s Research Hospital under relevant institutional and American Veterinary Medical Association (AVMA) guidelines and were performed in a biosafety level 2 facility that is accredited by the American Association for Laboratory Animal Science (AALAS).

### Mice.

Adult (6-week-old) female BALB/cJ mice were obtained from Jackson Laboratories (Bar Harbor, ME). Mice were housed in groups of 5 in high-temperature 31.2-cm by 23.5-cm by 15.2-cm polycarbonate cages with isolator lids. Rooms used for housing animals were maintained on a 12-h/12-h light/dark cycle at 22 ± 2°C with 50% humidity in the biosafety level 2 facility at St. Jude Children’s Research Hospital (Memphis, TN). Prior to inclusion in the experiments, mice were allowed at least 7 days to acclimate to the animal facility, such that they were 7 weeks old at the time of infection. Laboratory Autoclavable Rodent Diet (PMI Nutrition International, St. Louis, MO) and autoclaved water were available *ad libitum*. All experiments were performed under an approved protocol and in accordance with the guidelines set forth by the Animal Care and Use Committee at St. Jude Children’s Research Hospital.

### Tn-Seq.

Plasmid DNA harboring *magellan6*, a derivative of the Himar1 Mariner transposon, was purified from Escherichia coli with the Qiagen mini plasmid preparation kit (Qiagen). Pneumococcal DNA was isolated by phenol-chloroform extraction and ethanol precipitation from an exponentially growing culture in THY medium (30 mg/ml Todd-Hewitt broth powder and 0.2 mg/ml yeast extract). *In vitro*, *magellan6* transposition reactions were carried out with purified MarC9 transposase, 1 μg of pneumococcal target DNA, and 1 μg of *magellan6* plasmid DNA. Reaction mixtures were incubated for 1 h at 30°C, inactivated for 20 min at 72°C, ethanol precipitated, and resuspended in gap repair buffer (50 mM Tris [pH 7.8], 10 mM MgCl_2_, 1 mM dithiothreitol [DTT], 100 nM deoxynucleoside triphosphates [dNTPs] and 50 ng bovine serum albumin [BSA]). Repair of transposition product gaps was performed with E. coli DNA ligase overnight at 16°C. Repaired transposition products were transformed into naturally competent pneumococcal strain D39. The following day, colonies were scraped off tryptic soy agar (TSA) plates supplemented with 3% sheep erythrocytes and 200 mg/ml spectinomycin (TSA-Spec), pooled into libraries of approximately 50,000 transformants/library, split up into multiple starter cultures, and stored at −20°C.

### Bacterial fitness by Tn-Seq.

Bacteria were collected from the blood and lungs of each infected mouse at 12 h pbi or 24 h pbi and plated on TSA-Spec plates as indicated below in “Lung and blood harvesting.” Later time points could not be examined due to insufficient numbers of surviving mice. Bacteria were incubated for 12 h at 37°C and then collected in THY medium and centrifuged at 500 × *g* and 4°C for 10 min. The medium supernatant was removed, and the pellets were stored at −20°C. Gene identification by Tn-Seq was performed as described previously (51, 52, 80). The samples at each of the three time points (preselection [inoculum {*t*_1_}] and postselection [after infection, 12 h {*t*_2_} or 24 h {*t*_3_} pbi]) were sequenced in rapid run mode on an Illumina HiSeq 2000 using the primers and cycle conditions in Table S1A. For each insertion, the fitness, *W_i_*, was calculated by comparing the fold expansion of the mutant relative to the rest of the population with the following equation (91):

$$W_{i}=\frac{\ln\left[N_{i}\left(t_{2,3}\right)d/N_{i}\left(t_{1}\right)\right]}{\ln\left\{\left[1-N_{i}\left(t_{2,3}\right)\right]d/\left[1-N_{i}\left(t_{2,3}\right)\right]\right\}}$$

where the mutant frequencies at time zero and harvest are *N_i_*(*t*_1_) and *N_i_*(*t*_2,3_), respectively. The expansion factor (*d*) accounts for bacterial growth during library selection. Additional details of the method are included in the supplemental material.

### Infectious agents.

All experiments were done using the mouse-adapted influenza virus A/Puerto Rico/8/34 (H1N1) (PR8) and type 2 pneumococcal strain D39 variants. The transposon mutant library used for infections was generated as described above in “Tn-Seq.” To generate the single-gene-deletion (SGD) mutants, genomic DNA was isolated from WT D39 as described above in “Tn-Seq” and used as the template for gene splicing by overhang extension (SOE) PCR (92, 93). In brief, regions flanking the D39 locus targeted for deletion and containing overhangs complementary to the erythromycin (ERM) resistance cassette (*ermB*) (labeled regions A and B) were generated using the primer sets and cycle conditions indicated in Table S1B (EasyA PCR kit [Agilent Tech] and Bio-Rad T100 thermal cycler). Products were purified using the Zymoclean gel DNA recovery kit (Zymo Research), recombined by PCR (TaKaRa PCR kit [Clontech Laboratories]) using the primers and cycle conditions in Table S1C, and transformed into D39. Transformed bacteria were selected after overnight growth on TSA plates containing 1 μg/ml ERM (TSA-ERM). Infection stocks were grown in THY medium containing 1 μg/ml ERM and stored at −80°C in 12% glycerol. ERM resistance cassette insertion and target locus deletion were confirmed by PCR with the primer sets in Table S1D.

### Infection experiments.

The viral infectious dose (TCID_50_) was determined by interpolation using the method of Reed and Muench (94) using serial dilutions of virus on Madin-Darby canine kidney (MDCK) cells. Bacterial infectious doses (CFU) were determined by serial dilutions on TSA (WT), TSA-ERM (SGD mutants), or TSA-Spec (Tn-seq) plates. Frozen stocks of inocula were diluted in sterile PBS and administered intranasally to groups of 5 (for kinetics) or 10 (for bacteria collection and survival) mice lightly anesthetized with 2.5% inhaled isoflurane (Baxter, Deerfield, IL) in a total volume of 100 μl (50 μl per nostril). Mice were inoculated with either PBS or 75 TCID_50_ PR8 at day zero and then with 10^6^ CFU of the transposon mutant library, D39, or an SGD mutant (in 100 μl) 7 days later. This dose of bacteria was chosen to ensure that sufficient amounts of bacteria were recovered in Tn-Seq experiments, and the CFU of each inoculum was confirmed as described above. For bacterial sequencing, 500 μl of the inoculum was plated on TSA-Spec plates (100 μl/plate) (*t*_1_ in “Bacterial fitness by Tn-Seq”). Animals were weighed at the onset of infection and each subsequent day to monitor illness and mortality. Mice were euthanized if they became moribund or lost 30% of their starting body weight.

### Lung and blood harvesting.

Mice were euthanized by CO_2_ asphyxiation. For bacterial sequencing, lungs were perfused with 10 ml PBS, aseptically harvested, washed three times in PBS, and placed on ice in 500 μl PBS. The postperfusion fluid (mixture of blood and PBS) was plated immediately on TSA-Spec plates (150 μl/plate). Lungs were then enzyme digested with collagenase (1 mg/ml; Sigma) and physically homogenized using a syringe plunger against a 40-μm cell strainer. Cell suspensions were centrifuged at 500 × *g* and 4°C for 7 min, and the supernatant was plated on TSA-spec plates (500 μl; 100 μl/plate). For *in vitro* SGD growth, lungs were homogenized (Omni TH-01 with 5-mm flat blade) and centrifuged at 500 × *g* and 4°C for 7 min. For *in vivo* kinetics, unperfused lungs were enzyme digested with collagenase as described above and supernatants were used to quantify viral titers, bacterial titers, and cytokine/chemokine levels (5 mice/group). Following red blood cell lysis, cells were washed in staining buffer (PBS, 0.01 M HEPES, 5 mM EDTA, and 0.5% bovine serum albumin [BSA]), counted with trypan blue exclusion using a Cell Countess system (Invitrogen, Grand Island, NY), and prepared for flow cytometric analysis as described below.

### *In vitro* kinetics.

Bacteria were grown at 37°C in 1.0 ml of THY medium, PBS, or lung homogenate supernatants (s/n). At each time point, 50 μl was removed, serially diluted in PBS, and plated on TSA (WT) or TSA-ERM plates. Bacterial titers were normalized to the total volume. Three biological replicates were performed.

### Lung and blood titers.

For each mouse, viral titers were obtained using serial dilutions on MDCK monolayers, and bacterial titers were obtained using serial dilutions on TSA (WT) or TSA-ERM (SGD mutants) plates.

### Cytokines.

Cytokines and chemokines were measured in lung supernatant by using a Milliplex Luminex assay [GM-CSF, IFN-γ, IL-1α, IL-1β, IL-2, IL-6, IL-10, IL-12(p40), IL-12(p70), KC, MCP-1, MIP-1α, MIP-1β, RANTES, and TNF-α] and enzyme-linked immunosorbent assay (ELISA) (IFN-α,β). Prior to use, cell debris and aggregates were removed by centrifugation at 400 × *g* and 4°C. Milliplex magnetic bead cytokine/chemokine plates (Millipore) were prepared according to the manufacturer’s instructions. Analysis was done using a Bio-Rad BioPlex (HTF system) and Luminex xPonent software. ELISAs for IFN-α and IFN-β (PBL Assay Science) were prepared according to the manufacturer’s instructions. Plates were read at 450 nm and analyzed using the website elisaanalysis.com (no longer available) and confirmed in Prism v9.1.0. The mean concentrations of duplicate samples were calculated by construction of standard curves using a weighted 5PL and 4PL regression for the Milliplex and ELISA data, respectively. Absolute quantities of each cytokine/chemokine were calculated based on the mean concentrations of replicate samples normalized to the lung supernatant volume collected during tissue processing.

### Flow cytometric analysis.

Flow cytometry (LSRII Fortessa; Becton, Dickinson, San Jose, CA) was performed on single cell suspensions after incubation with 200 μl of a 1:2 dilution of Fc block (human gamma globulin) on ice for 30 min, followed by surface marker staining with anti-mouse antibodies to the following proteins: CD11c (clone N418, eFluor450; eBioscience), CD11b (clone M1/70, Alexa Fluor 700; BD Biosciences), Ly6G (clone 1A8, peridinin chlorophyll protein [PerCp]-Cy5.5; Biolegend), Ly6C (clone HK1.4, allophycocyanin [APC]; eBioscience), F4/80 (clone BM8, phycoerythrin [PE]; eBioscience), CD3e (clone 145-2C11, PE-Cy7 [BD Biosciences] or BV785 [Biolegend]), CD4 (clone RM4-5, PE-Cy5; BD Biosciences), CD8α (clone 53-6.7, BV605; BD Biosciences), CD49b (clone DX5, APC-Cy7 [Biolegend] or APC-e780 [Affymetrix, Inc.]), and major histocompatibility complex class II (MHC-II) (clone M5/114.15.2, fluorescein isothiocyanate [FITC]; eBioscience). The data were analyzed using FlowJo 10.4.2 (Tree Star, Ashland, OR), where viable cells were gated from a forward scatter/side scatter plot and singlet inclusion. Following neutrophil exclusion (Ly6G^hi^), macrophages (MΦ) were gated as CD11c^hi^ F4/80^hi^, with alveolar macrophages (AMΦ) subgated as CD11b^−^ and inflammatory macrophages (IMΦ) as CD11b^+^. After macrophage exclusion, T cell populations were gated as CD3e^+^ and subgated into CD8 T cells (CD3^+^ CD8^+^ CD4^−^ DX5^−^) and CD4 T cells (CD3^+^ CD8^−^ CD4^+^ DX5^−^). From the CD3e^−^ population, natural killer (NK) cells were gated as CD3^−^ DX5^+^ and dendritic cells (DCs) as CD3^−^ DX5^−^. DCs were further gated into three subsets of DCs: CD11c^+^ CD11b^−^, CD11c^+^ CD11b^+^, and CD11c^−^ CD11b^+^ (Fig. S5). The expression level of MHC-II was used to confirm the identities and activation of MΦ and DC subsets. The absolute numbers of cell types were calculated based on viable events analyzed by flow cytometry and normalized to the total number of viable cells per sample.

### Histology.

Mice were euthanized by CO_2_ asphyxiation, and lungs were inflated *in situ* via tracheal infusion with 10% neutral buffered formalin solution (NBF; ThermoFisher Scientific, Waltham, MA), followed by continued fixation in NBF for at least 2 weeks before being embedded in paraffin, sectioned at 4 μm, mounted on positively charged glass slides (Superfrost Plus; Thermo Fisher Scientific, Waltham, MA), and dried at 60°C for 20 min. Tissue sections were stained with hematoxylin and eosin (H&E) or subjected to immunohistochemical (IHC) staining to detect influenza virus antigen, pneumococcus, and neutrophils. For detection of these targets, tissue sections underwent antigen retrieval in a prediluted cell conditioning solution (CC1) (catalog number 950-124; Ventana Medical Systems, Indianapolis, IN) for 32 min on a Discovery ultra immunostainer (Ventana Medical Systems, Tucson, AZ). Primary antibodies included (i) a polyclonal goat antibody raised against the hemagglutinin (HA) glycoprotein of B/Florida/04/2006 (Yamagata lineage) influenza virus diluted 1:2,000 (catalog number I7650-05G; US Biologicals, Swampscott, MA), (ii) a rabbit polyclonal antibody to Streptococcus pneumoniae diluted 1:1,000 (catalog number NB100-64502; Novus Biologicals, Littleton, CO), and (iii) a rat monoclonal antibody to neutrophils (Ly6G6C) diluted 1:50 (catalog number NB600-1387; Novus Biologicals, Littleton, CO). Binding was detected using OmniMap anti-goat (#760-4647), anti-rabbit (#760-4311), and anti-rat (#760-4457) secondary antibody-horseradish peroxidase (HRP), respectively (Ventana Medical Systems), with the Discovery ChromoMap 3,3′-diaminobenzidine (DAB) kit (Ventana Medical Systems) as the chromogenic substrate. Stained sections were examined by a pathologist (P.V.) who was blind to the experimental group assignments. IHC data were quantified (A.P.S.) using the immunohistochemistry recipe number L006-02 provided in MIPAR, version 3.0.

### Statistical analysis.

Significant differences in Kaplan-Meier survival curves were calculated using the log rank test. *In vitro* growth/decay rates were analyzed by nonlinear regression of log_10_ values, and linear slopes were compared by analysis of covariance (ANCOVA). The remainder of the statistical analyses were performed on linear values. Unpaired *t* tests were done to analyze *in vitro* growth dynamics in THY medium and lung cultures. Analyses of variance (ANOVA) were performed using a Dunnett correction for multiple comparisons (to WT D39) to analyze *in vivo* differences, including lung and blood bacterial loads, viral loads, immune cells, cytokines, and chemokines (GraphPad Prism 7.0c). The confidence interval of significance was set to 95%, and *P* values of less than 0.05 were considered significant.

### Data availability.

Illumina sequence reads have been deposited in the NCBI Sequence Read Archive (http://www.ncbi.nlm.nih.gov/sra) under accession number PRJNA727261.
